# Celastrol improves endothelial function in diet-induced obesity mice via attenuating endoplasmic reticulum stress through the activation of AMPK pathway

**DOI:** 10.1186/s10020-025-01259-6

**Published:** 2025-06-11

**Authors:** Cheng Yu, Weihong Lin, Jing Yang, Qiong Jiang, Wenkun Liu, Hongjin Liu, Yong Lin, Litao Wang, Lei Chen, Yu Huang, Lianglong Chen

**Affiliations:** 1 Department of Cardiology, Fujian Medical University Union Hospital, Fujian Cardiovascular Medical Center, Fujian Institute of Coronary Artery Disease, Fujian Cardiovascular Research Center, Fuzhou, Fujian 350001 P. R. China; 2 Department of Cardiovascular Surgery, Fujian Medical University Union Hospital, Fuzhou, Fujian 350001 China

**Keywords:** Celastrol, Endoplasmic reticulum stress, Obesity, Endothelial function, AMPK

## Abstract

**Background:**

Diet-induced obesity (DIO) is a significant factor in endothelial dysfunction. Celastrol, a potent anti-inflammatory and anti-oxidative pentacyclic triterpene, has shown promise as a protective agent against cardiovascular disease. However, the specific protective effects and mechanisms of celastrol in preventing endothelial dysfunction in diet-induced obesity are not yet fully understood.

**Methods and results:**

In this study, eight-week-old C57BL/6 mice were fed a normal or high-fat diet and treated with or without celastrol for 8 weeks. We measured acetylcholine-induced endothelium-dependent relaxation (EDR) in the aortae using a wire myograph. The results revealed that EDR was impaired in DIO mice, along with decreased AMPK phosphorylation, increased endoplasmic reticulum (ER) stress, and reactive oxygen species (ROS) in the aortae. These effects were reversed by celastrol treatment. Celastrol also reversed tunicamycin-induced ER stress, decreased nitric oxide (NO) production, and impaired EDR in mouse aortae. The protective effects of celastrol were negated by co-treatment with an AMPK inhibitor (Compound C). Furthermore, in AMPKα deficient mice, the beneficial effects of celastrol on EDR were significantly reduced.

**Conclusions:**

These findings suggest that celastrol improves endothelial function by inhibiting ER stress and increasing NO production through the activation of the AMPK pathway in DIO mice.

**Graphical Abstract:**

The schematic diagram illustrates the mechanism by which celastrol ameliorates endothelial-dependent vasodilatation in diet-induced obesity mice. Celastrol activates the AMPK signaling pathway, thereby suppressing endoplasmic reticulum (ER) stress, inflammation, and reactive oxygen species (ROS) generation, which collectively enhance endothelial-dependent vasodilatation in diet-induced obesity mice.

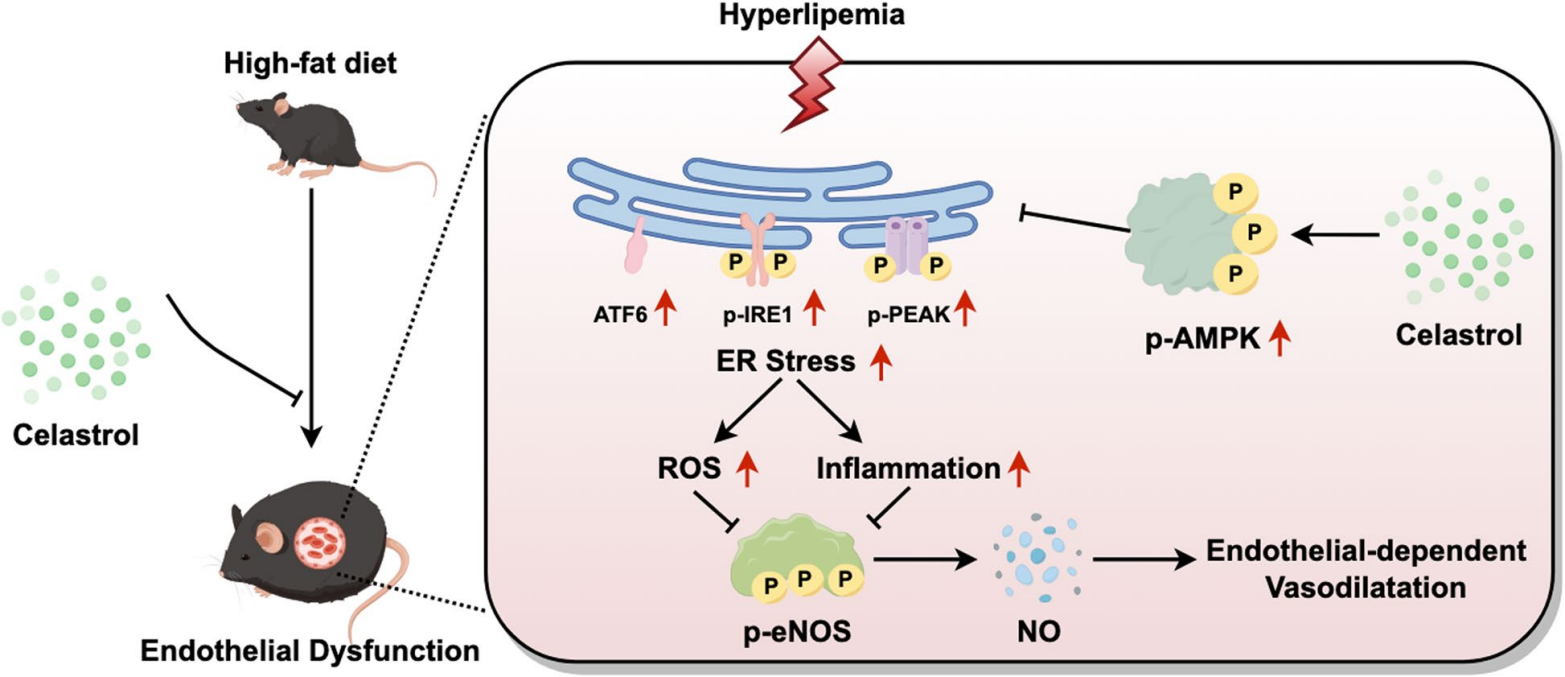

**Supplementary Information:**

The online version contains supplementary material available at 10.1186/s10020-025-01259-6.

## Introduction

Obesity is a significant global public health issue, ranking as the fourth leading cause of death following hypertension, dietary risks, and tobacco use (Balakrishnan 2022). This multifaceted disease is linked to various non-communicable diseases like cardiovascular disease, type 2 diabetes mellitus, and cancer, all of which impact both quality and length of life (Blüher 2019). Current research is focused on effectively reducing complications associated with obesity (Perdomo et al. 2023).

Studies have demonstrated that endothelial dysfunction is present in both obese patients and animal models, often serving as a precursor to cardiovascular conditions like hypertension and atherosclerosis (Koenen et al. 2021). Endothelial-dependent relaxation (EDR) impairment, a key indicator of endothelial dysfunction, is linked to decreased nitric oxide (NO) levels and an increase in vasoconstrictive substances such as angiotensin II, noradrenaline, and endothelin-1 (Syed et al. 2022). Many studies suggest that diet-induced obesity is associated with reduced NO availability (Ionică et al. 2021), highlighting the importance of restoring NO levels as a therapeutic approach for endothelial dysfunction.

Emerging evidence from experimental research suggests that endoplasmic reticulum (ER) stress plays a significant role in decreasing NO bioavailability (Zachariah et al. 2021). The ER is a crucial organelle responsible for the folding and assembly of secretory and membrane proteins. Disruption of this function leads to the accumulation of unfolded proteins in the ER, causing ER stress (Oakes and Papa 2015). It has been demonstrated that ER stress leads to endothelial dysfunction in aortae and mesenteric resistance arteries, and inhibition of ER stress with tauro-ursodeoxycholic acid (Tudca) or 4-Phenylbutyric acid (4-PBA) improves the EDR in obese and spontaneous hypertensive rats (Galán et al. 2014; Cheang et al. 2014; Naiel et al. 2019). Celastrol, a bioactive compound derived from Tripterygium wilfordii and Cilantros orbiculatus, is commonly used in the clinical treatment of chronic inflammatory and immune disorders (Lei et al. 2025). Apart from immune system, emerging evidence began to support varying protective roles celastrol in cardiovascular diseases such as anti-arrhythmia (Tan et al. 2024), diabetic or hypertrophic cardiomyopathy (Zhao et al. 2023), and ischemia-induced cardiac fibrosis (Fan et al. 2023). In addition, the recently identified anti-obesity property of celastrol adds more complexity to its biofunction (Kusminski et al. 2024; Zhao et al. 2024). Specifically, its therapeutic potential in obesity has been supported in hepatic steatosis and DIO mice through targeting adipocytes (Li et al. 2025; Ouyang et al. 2024). However, whether cardiovascular benefits of celastrol, especially improvements in endothelial function were attributed to its anti-obesity action still remains elusive. Therefore, this study aims to investigate the protective effects of celastrol on endothelial function in DIO mice by inhibiting ER stress through the activation of the AMPK pathway.

## Materials and methods

### Animals and treatments

The study was approved by the Animal Research Ethics Committee of Fujian Medical University and adhered to the Guide for the Care and Use of Laboratory Animals by the National Institutes of Health. Male C57BL/6 J mice aged 8 weeks were divided into four groups randomly: Control treated with saline (*n* = 6); (2) Control treated with celastrol (*n* = 6); (3) DIO treated with saline (*n* = 6); (4) DIO treated with celastrol (*n* = 6) (Figure S1 A). These mice were fed either a standard diet or a high-fat diet comprising 45 kcal% fat for 8 weeks to induce obesity. Additionally, the mice received intraperitoneal administration of celastrol (100 μg/kg/day, MedChemExpress) or saline for the same duration. Endothelial cell (EC)-specific AMPKα knockout (KO) mice were generated by cross-breeding AMPKα-LoxP mice and *Cdh5*-Cre mice. 8-week-old male AMPKα-loxP/*Cdh5*-Cre mice and AMPKα-loxP mice were treated with tamoxifen at 75 mg/kg for 5 consecutive days with 2 days break in-between to induce EC-specific AMPKα knockout (AMPKα^EC−/−^). All mice were maintained in standard cages in a specific pathogen-free environment, with a 12-h light/dark cycle, and had access to food and water ad libitum.

Blood pressures were indirectly measured at 16 weeks of age using a tail-cuff method (BP-98 A; Softron, Tokyo, Japan) as described previously (Yu et al. 2021). Plasma glucose levels were quantified using a glucose monitor (Accu-chek, Roche, France). Total cholesterol and triglyceride levels in plasma were determined by enzyme immunoassay (Beyotime Biotechnology, Shanghai, China).

### Histological analysis

Liver tissues were formalin-fixed and embedded in paraffin and cut into 5 μm sections stained with hematoxylin & eosin (H&E). Frozen liver sections embedded in the O.C.T. compound were stained with Oil Red O, and the red lipid droplets were visualized using a Nikon microscope (Nikon, Melville, NY, USA). Adipocyte size was measured using ImageJ software based on six sections per mouse.

### Inflammatory markers measurement

The IL-1β, IL-10, and TNF-α levels in thoracic aortae tissue were measured by enzyme-linked immunosorbent assay (ELISA) using a commercially available kit (Boster, Wuhan, China) according to the manufacturer’s protocol.

### Functional assay by wire myograph

After mice were sacrificed, thoracic aortae were dissected and cleaned of adhering connective tissue using oxygenated ice-cold Krebs solution as previously described (Yu et al. 2021). The aortic rings, approximately 2 mm in length, were mounted on a myograph (Danish Myo Technology, Aarhus, Denmark) for isometric tension recording. The rings were stretched to an optimal baseline tension and allowed to equilibrate for 60 min. Contraction was induced using KCl (60 mmol/L) followed by rinsing in Krebs solution. The contraction of aortic rings was determined by the cumulative addition of phenylephrine (PE, 10^–9^−10^–5^ M; Sigma-Aldrich). EDR was determined by the cumulative addition of acetylcholine (Ach, 10^–8.5^–10^−5^ M; Sigma) and endothelium-independent relaxation were determined by the cumulative addition of sodium nitroprusside (SNP, 10^–9^−10^–5^ M; Sigma-Aldrich) in PE (3 µmol/L; Sigma) pre-contracted rings. To investigate the role of eNOS in the EDR studies, the aortic rings were incubated with L-NAME (100 μM) for 30 min before assessing the effects on Ach-induced relaxation.

### Ex vivo studies of aortic rings

Aortic rings were dissected and incubated in Dulbecco’s Modified Eagle’s Media (DMEM, Gibco) with 10% fetal bovine serum (Gibco). Tunicamycin (ER stress inducer, 2 mg/mL; Sigma), celastrol (1 nmol/L; MedChemExpress), or Compound C (AMPK antagonist, 5 mmol/L; Sigma-Aldrich) was added into the culture medium that bathed the aortic rings in an incubator at 37℃ for 16 h. After the incubation, the rings were transferred into fresh Krebs solution for functional studies in myograph and western blotting.

### ROS measurement

Aortic rings were frozen, sliced into 10 μm sections using a Leica CM 100 cryostat, and then incubated with the fluorescent dye dihydroethidium (DHE, 5 mmol/L; Invitrogen) in normal physiological saline solution for 15 min at 37℃. Fluorescence images were captured with the Olympus Fluoview FV1000 laser scanning confocal system.

### Measurement of NO metabolites

The aortic rings were stimulated with Ach (10^–6^ M) for 5 min, then dabbed dry with filter paper and weighed. The incubation solution was assayed for the stable end-product of NO, that is, nitrate (NO_3–_) and nitrite (NO_2–_), using the nitrate reductase method (Nanjing Jiancheng Bioengineering Institute, Nanjing, China) as manufacturer’s instructions.

### Western blotting analysis

Protein samples were separated by 10% SDS-PAGE and electro-transferred to PVDF membranes. Membranes were blocked and incubated with specific primary antibodies against p-AMPKα at Thr^172^, p-PERK at Thr^980^, p-IRE1 at Ser^724^, ATF6, p-eNOS at Ser^1177^, p-LKB1 at Ser^428^, p-ACC at Ser^79^, NOX2, iNOS, t-AMPKα, t-PERK, t-IRE1, t-LKB1, t- ACC, GAPDH (1:1000, Proteintech). Immunoreactive bands were detected by incubating with secondary antibodies conjugated to horseradish peroxidase and enhanced chemiluminescence reagent. The band intensities were quantified by densitometry using Quantity-One software (Bio-Rad, Hercules, CA), and normalized with GAPDH expression.

### Statistical analysis

Results represent means ± SD from different groups. The relaxation was presented as a percentage reduction of the PE-induced contraction. Data were analyzed using GraphPad Prism 10.0 software. Comparisons among groups were made using ANOVA followed by Sidak’s or Newman-Keuls test. The results were considered statistically significant with *P* values < 0.05.

## Results

### Effect of celastrol on general parameters in DIO mice

**Fig. 1:**
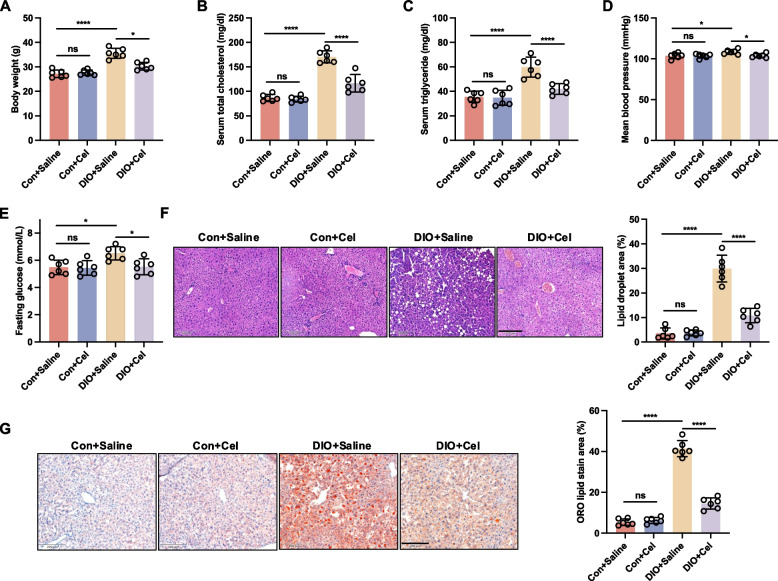
Celastrol emerged as an anti-obesity agent in diet-induced obesity (DIO) mice. DIO and control (Con) mice were treated with saline or celastrol (Cel, 100 μg/kg/day) intraperitoneally (i.p) for 8 weeks. **A** Body weight, **B** total cholesterol, **C** triglyceride, **D** blood pressure, and (**E**) blood glucose were measured during the treatment. **F** Hematoxylin & eosin (H&E) and (**G**) Oil Red O (ORO) staining of liver sections from DIO and Con mice treated with saline or celastrol for 8 weeks, Scale bar, 200 μm. Data are expressed as the means ± S.E.M (*n* = 6/group), ^*^P < 0.05, one-way ANOVA followed by Newman-keuls post hoc test

The body weight, total cholesterol, triglyceride, blood pressure, and blood glucose levels were significantly elevated in DIO mice compared to the control group (Fig. 1A-E and Fig. S1B). To investigate whether celastrol could prevent metabolic complications associated with obesity induced by a high-fat diet, DIO mice were treated with celastrol at a dosage of 100 μg/kg/day for 8 weeks. Following celastrol treatment, these above abnormal indicators were normalized (Fig. 1A-E). Histological analyses, including hematoxylin and eosin (H&E) and oil red O staining, demonstrated a reduction in hepatic lipid accumulation in the treated DIO mice (Fig. 1F and G). Overall, these data suggested that celastrol may alleviate high-fat induced metabolic complications.

### Celastrol rescued endothelial dysfunction in DIO mice

**Fig. 2:**
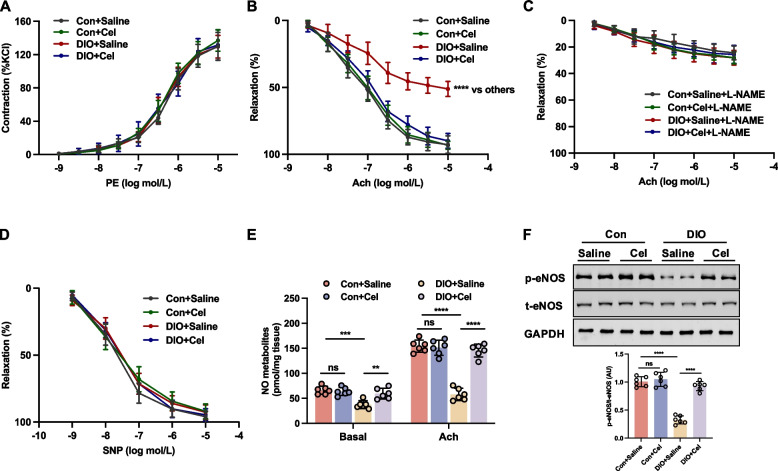
Celastrol rescued endothelial dysfunction in diet-induced obesity (DIO) mice. DIO and control (Con) mice were treated with saline or celastrol (Cel, 100 μg/kg/day) intraperitoneally (i.p) for 8 weeks. **A** PE-induced contraction. **B** Ach-induced relaxation. **C** Ach-induced relaxation in the absence or presence of L-NAME (100 µM). **D** SNP-induced relaxation. **E** Basal and Ach-stimulated NO production in endothelium-intact aortae. **F** Phosphorylation of eNOS at Ser^1177^ in aortae was analyzed by western blotting. Data are expressed as the means ± S.E.M (*n* = 6/group), ^*^P < 0.05, two-way ANOVA with Sidak’s post hoc analysis for A-D, one-way ANOVA followed by Newman-keuls post hoc test for **E** and **F**

To determine the potential protective effect of celastrol on vascular reactivity in DIO mice, aortic rings were treated with cumulative concentrations of PE (10^–9^−10^–5^ M) and KCl (125 mM). Results showed no significant difference in the contractile response induced by PE between the control and DIO groups, regardless of celastrol treatment (Fig. 2A). To evaluate the function of EDR, aortic rings were initially precontracted using PE stimulation, followed by treatment with Ach. Our findings indicate that Ach-induced relaxation was impaired in DIO compared to the control group, which was significantly reversed by celastrol treatment (Fig. 2B). Pretreatment with L-NAME (100 μM) inhibited Ach-induced relaxation in both the DIO and control groups, irrespective of celastrol treatment, resulting in no significant differences in Ach-induced relaxation among these groups (Fig. 2C). Impaired Ach-induced relaxation in aortic rings is associated with reduced NO production (Alikhani et al. 2022). Endothelium-independent relaxation was also measured in response to SNP (10^–9^−10^–5^ M), and no differences were observed across all groups, suggesting that the vascular smooth muscle response to NO was unaffected (Fig. 2D). To assess NO production, we measured nitrate/nitrite levels and evaluated endothelial nitric oxide synthase (eNOS) activity by measuring the phosphorylation of eNOS at Ser^1177^. Our findings indicate that NO production and eNOS phosphorylation at Ser^1177^ were diminished in DIO mice, but these reductions were ameliorated by celastrol treatment (Fig. 2E and F). Collectively, these results further demonstrated celastrol can ameliorate endothelial dysfunction but does not influence NO sensitivity, in DIO mice.

### Inhibition of ER stress contributed to the beneficial effect of celastrol on endothelial function in DIO mice

**Fig. 3:**
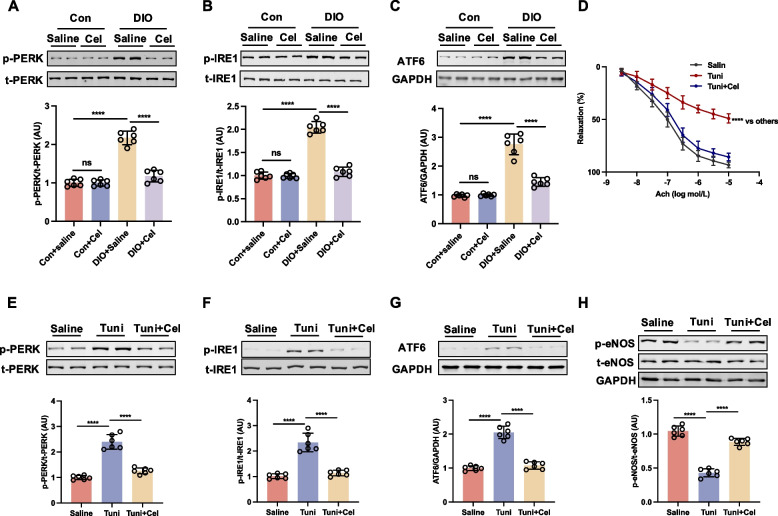
Celastrol improved endothelial function via ameliorating ER stress in diet-induced obesity (DIO) mice. DIO and control (Con) mice were treated with saline or celastrol (Cel, 100 μg/kg/day) intraperitoneally (i.p) for 8 weeks. **A**-**C** The expressions of ER stress markers, including p-PERK, ATF6, and p-IRE1 in aortae were analyzed by western blotting. The aortae from Con mice were incubated with tunicamycin (Tuni, 2 μg/mL), or celastrol (1 nmol/L) for 16 h. **D** Ach-induced relaxation and (**E–G**) the expressions of ER stress markers, including p-PERK, ATF6, and p-IRE1, and (**H**) the phosphorylation of eNOS at Ser^1177^ in aortae. Data are expressed as the means ± S.E.M (*n* = 6/group), ^*^P < 0.05, two-way ANOVA with Sidak’s post hoc analysis for **D**, one-way ANOVA followed by Newman-keuls post hoc test for others

ER stress arises from intricate intracellular signaling cascades encompassing three primary ER stress transduction pathways: PERK, ATF6, and IRE1 (Luo et al. 2022). Previous research has demonstrated a significant upregulation of ER stress in DIO mice (Wang et al. 2023). In alignment with previous findings, our study observed elevated levels of ER stress markers, specifically the phosphorylation of PERK and IRE1, as well as increased expression of ATF6, in the DIO group compared to the control group, which were reversed by treatment with celastrol for 8 weeks (Fig. 3A-C). To further pinpoint which pathway is responsible for celastrol-regulated ER stress, we tested the expression of their upstream regulator, BiP, and downstream effectors, ATF4, XBP1 s, and CHOP, respectively. We found the expression of BiP was decreased following celastrol treatment as accompanied by the decrease of the downstream effectors of PERK, IRE1, and ATF6, including ATF4, XBP1, and CHOP (Figure S2). Altogether, these results show that celastrol negatively regulates BiP, therefore comprehensively downregulating its effectors, ATF4, XBP1 and CHOP as a whole. To elucidate the role of inhibiting ER stress in enhancing endothelial function through celastrol, we investigated the impact of ER stress on endothelial dysfunction, induced by ex vivo exposure to tunicamycin (ER stress inducer). Co-incubation with celastrol ameliorated the tunicamycin-induced impairment of EDR in aortic rings from control mice (Fig. 3D). Moreover, we found that celastrol could significantly inhibit tunicamycin-induced ER stress, including decreased phosphorylation of PERK, IRE1, and the expressions of ATF-6 and increased tunicamycin-induced the reduction of phosphorylation of eNOS at Ser^1177^ in aortic rings from control mice (Fig. 3E-H). Therefore, these results demonstrated that celastrol repressed high-fat induced ER stress, thereby enhancing endothelial function in DIO mice.

### Celastrol ameliorated ER stress dependent on AMPK signaling in DIO mice and aortic rings incubated with tunicamycin

**Fig. 4:**
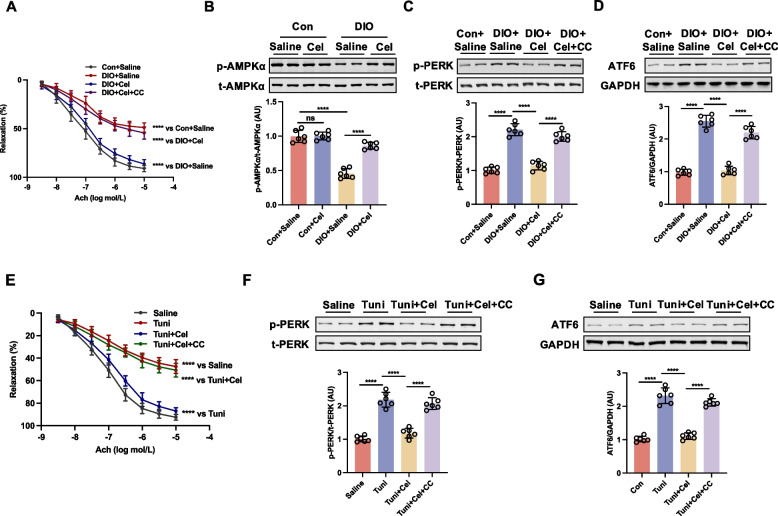
Celastrol ameliorated ER stress dependent on the activation of AMPK in diet-induced obesity (DIO) mice and aortic rings incubated with tunicamycin. The aortae from DIO and control (Con) treated with saline or celastrol (Cel, 100 μg/kg/day) intraperitoneally (i.p) for 8 weeks were incubated with or without Compound C (CC, 5 μmol/L) for 16 h. **A** Ach-induced relaxation, (**B-D**) the phosphorylation of AMPKα and the expressions of ER stress markers, including p-PERK and ATF6 in aortae were analyzed by western blotting. The aortae from control mice were incubated with tunicamycin (Tuni, 2 μg/mL), Compound C (5 μmol/L) or celastrol (1 nmol/L) for 16 h. **E** Ach-induced relaxation and (**F **and** G**) the expressions of ER stress markers, including p-PERK and ATF6 in aortae were analyzed by western blotting. Data are expressed as the means ± S.E.M (*n* = 6/group), ^*^P < 0.05, two-way ANOVA with Sidak’s post hoc analysis for A and E, one-way ANOVA followed by Newman-keuls post hoc test for **B**-**D**, **F**, and **G**

Previous research has demonstrated that AMP-activated protein kinase (AMPK) ameliorates ER stress, suggesting that ER stress operates downstream of AMPK (Sang et al. 2022). To investigate whether celastrol ameliorated ER stress through AMPK signaling, aortic rings from DIO mice treated with celastrol were incubated with the AMPK inhibitor Compound C. The inhibition of AMPK by Compound C abrogated the celastrol-induced improvement in EDR in DIO mice (Fig. 4A). Furthermore, we observed that the phosphorylation level of AMPKα at Thr^172^, p-LKB1, as upstream regulator of AMPK and p-ACC, as a downstream target of AMPK were reduced in DIO mice; this reduction was reversed following an 8-week treatment with celastrol (Fig. 4B and Fig. S3). Celastrol has been shown to significantly inhibit ER stress, as evidenced by decreased phosphorylation of PERK and reduced expression of ATF-6 in DIO mice, an effect that was mitigated by treatment with Compound C (Fig. 4C-D). The interplay between AMPK and ER stress was further elucidated through ex vivo experiments, wherein aortic rings from control mice were exposed to tunicamycin followed by celastrol, with or without Compound C, for 16 h. Tunicamycin exposure diminished acetylcholine (Ach)-induced relaxation in aortic rings, an effect that was reversed by celastrol treatment. However, the presence of Compound C abrogated the restorative effect of celastrol (Fig. 4E). Moreover, celastrol demonstrated a significant inhibitory effect on tunicamycin-induced ER stress, as evidenced by reduced phosphorylation of PERK and decreased expression of ATF-6. Conversely, Compound C abrogated the capacity of celastrol to mitigate ER stress (Fig. 4F-G). These findings suggest that the amelioration of ER stress by celastrol, which contributes to improved endothelial function in DIO mice, is contingent upon the activation of AMPK.

### Celastrol alleviates ER stress-associated inflammation and oxidative stress via AMPK signaling in DIO mice

**Fig. 5:**
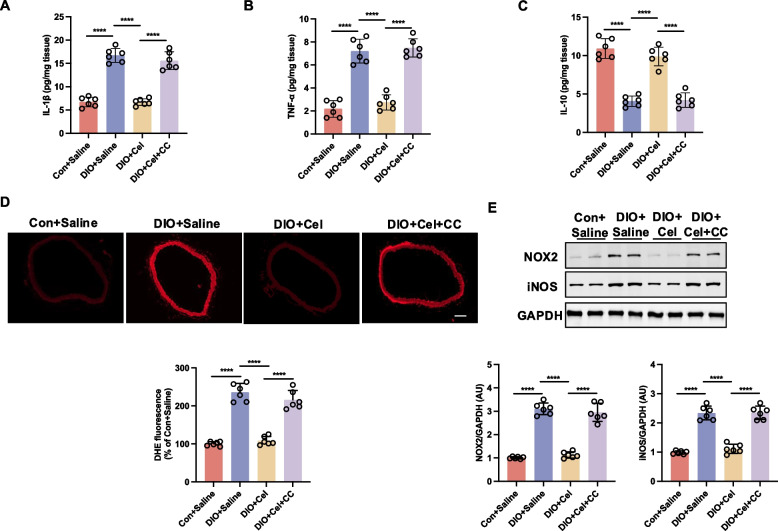
Celastrol alleviates ER stress-associated inflammation and oxidative stress via AMPK signaling in diet-induced obesity (DIO) mice. The aortae from DIO and control (Con) treated with saline or celastrol (Cel, 100 μg/kg/day) intraperitoneally (i.p) for 8 weeks were incubated with or without Compound C (CC, 5 μmol/L) for 16 h. **A-C** The inflammatory markers, including IL-1β, TNF-α, and IL-10 in thoracic aortae tissue, were measured by ELISA; (**D**) Representative images and quantified dihydroethidium (DHE) fluorescence in mice aortae, Scale bar, 100 μm; (**E**) Protein expressions of Nox-2 and iNOS in aortae were analyzed by western blotting. Data are expressed as the means ± S.E.M (*n* = 6/group), ^*^P < 0.05, one-way ANOVA followed by Newman-keuls post hoc test

ER stress has been reported to initiate a burst of inflammation and oxidative stress (Li et al. 2020). We investigated whether celastrol could reduce inflammation and oxidative stress in DIO mice. Our findings indicated that, in comparison to the control group, the DIO mice exhibited elevated levels of pro-inflammatory markers, such as IL-1β and TNF-α, and reduced levels of the anti-inflammatory marker IL-10 (Fig. 5A-C). Celastrol treatment could significantly reverse the abnormal levels of the above inflammatory markers in DIO mice. However, the administration of Compound C inhibited celastrol efficacy in reducing the inflammatory levels within the thoracic aortae tissue of DIO mice (Fig. 5A-C). In alignment with the observed inflammatory changes, oxidative stress levels were also elevated in DIO mice. Specifically, the thoracic aortae of DIO mice exhibited increased reactive oxygen species (ROS) production, as well as elevated expression of NOX2 and inducible nitric oxide synthase (iNOS), compared to control mice (Fig. 5D and E). Celastrol treatment also reduced the oxidative stress in DIO, while the administration of Compound C inhibited the ability of celastrol to attenuate oxidative stress in the thoracic aortae tissue of DIO mice (Fig. 5D and E). These results indicate that celastrol plays a crucial role in modulating inflammation and oxidative stress in DIO mice.

### AMPKα deficiency abolished the improvement effect of celastrol on endothelial function in mice aortae

**Fig. 6:**
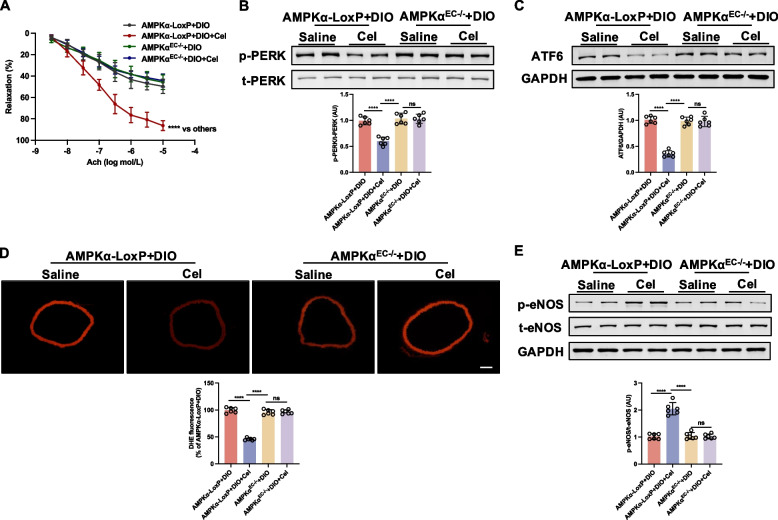
Endothelial cell-specific AMPKα knockout abolished the improvement effect of celastrol on endothelial function in diet-induced obesity (DIO) mice aortae. AMPKα-LoxP and AMPKα^EC−/−^ mice fed a high-fat diet were treated with saline or celastrol (Cel, 100 μg/kg/day) intraperitoneally (i.p) for 8 weeks. **A** Ach-induced relaxation, (**B **and** C**) the expressions of ER stress markers, including p-PERK and ATF6, **C** dihydroethidium (DHE) fluorescence images, and (**E**) phosphorylation of eNOS at Ser^1177^ in aortae. Data are expressed as the means ± S.E.M (*n* = 6/group). ^*^P < 0.05, two-way ANOVA with Sidak’s post hoc analysis for **A**, one-way ANOVA followed by Newman-keuls post hoc test for **B**-**E**

In aortae from DIO AMPKα-LoxP mice, celastrol treatment restored impaired EDR, an effect not observed in endothelial cell-specific AMPKα knockout (AMPKα^EC−/−^) (Fig. 6A). Moreover, indicators of ER stress, including phosphorylated PERK and ATF-6 expression, were increased in aortae from both DIO AMPKα-LoxP and AMPKα^EC−/−^ mice but were diminished in DIO AMPKα-LoxP mice following celastrol treatment (Fig. 6B-C). Furthermore, celastrol treatment resulted in a reduction of the superoxide level in DIO AMPKα-LoxP mice but was not observed in DIO AMPKα^EC−/−^ mice (Fig. 6D). In aortae from DIO AMPKα-LoxP mice, phosphorylation of eNOS at Ser^1177^ was reduced, whereas celastrol treatment promoted an increase in eNOS phosphorylation (Fig. 6E); while celastrol failed to enhance eNOS phosphorylation in aortae from DIO AMPKα^EC−/−^ mice. Collectively, these data suggest that the deficiency of AMPKα abolished the improvement effect of celastrol on endothelial function.

## Discussion

The present study demonstrated the critical role of celastrol in inhibiting ER stress and protecting endothelial function by AMPK activation in DIO mice. Our observations indicated that the impaired endothelium-dependent relaxation, activation of ER stress, and increased ROS production in the aortae of DIO mice were effectively reversed following chronic administration of celastrol.

Extensive research has demonstrated that the incidence of cardiovascular and cerebrovascular diseases is markedly higher in obese patients compared to non-obese individuals, with vascular endothelial dysfunction identified as the initiating factor (Koenen et al. 2021). Vascular endothelium is crucial for ensuring smooth arterial blood flow and maintaining the normal contractile and relaxation functions of blood vessels, which relies on the real-time"monitoring"of blood flow within the vessels (Augustin and Koh 2024). Furthermore, the endothelium plays a crucial role in protecting tissues and organs from a range of toxic substances by modulating the transport of various molecules between blood vessels and surrounding tissues and organs (Cheng and Huang 2024). Once the vascular endothelial function is compromised, local blood vessels become susceptible to thrombosis and plaque formation, which subsequently serve as potential risk factors for atherosclerosis (Hooglugt et al. 2022). Simultaneously, it can contribute to vascular smooth muscle remodeling and induce abnormal vasoconstriction and diastolic function, ultimately resulting in hypertension (Tomiyama 2023). Consequently, ameliorating endothelial dysfunction in obese patients is crucial for mitigating obesity-related complications.

Several mechanisms associated with endothelial dysfunction have been reported, including increased oxidative stress, inflammation, the dysregulation of the sympathetic nervous system and/or the renin-angiotensin system (Incalza et al. 2018; Della Corte et al. 2016; Jiang et al. 2022; Noureddine et al. 2024). It was also reported that ER stress is an important pathophysiological mechanism of endothelial dysfunction (Luo et al. 2022). ER stress is upregulated in the aortae of spontaneously hypertensive rats and obesity mice, contributing to vascular endothelial dysfunction (Liu et al. 2023a; Cheang et al. 2017). Our observations are in line with previous studies reporting that ER stress was upregulated in aortae from diet-induced obesity mice, which was accompanied by impaired endothelial function. Given PERK, IRE1 and ATF6 are critical regulators of ER stress, we tested the expression of their downstream effectors, ATF4, XBP1, and CHOP, respectively, following celastrol treatment. Consequently, we found all of them were decreased in response to celastrol treatment. Of note, BiP, the upstream regulator of PERK, IRE1, and ATF6, was also concomitantly attenuated. Altogether, these results showed that celastrol negatively regulated ER stress via BiP. ER stress initiates a burst of oxidative stress in the ER lumen and by targeting the mitochondria, triggers the elevation of ROS production (Li et al. 2020; Wei et al. 2022). Meanwhile, ER stress has been shown to activate the NF-κB and NLRP3 inflammatory pathways, thereby promoting the inflammatory cascade (Shaito et al. 2022). The excessive production of ROS leads to a decrease in NO bioavailability, adversely impacting endothelial function (Guo et al. 2023). In chronic inflammation, iNOS continuously produces a large amount of NO, which combines with O₂⁻ to produce peroxynitrite (ONOO⁻), leading to protein nitration, DNA damage, and mitochondrial dysfunction (Liu et al. 2023b). eNOS maintains vascular homeostasis by releasing NO in low concentration and transient state but is vulnerable to oxidative stress (Srinivasan et al. 2019). Similarly, our observations indicated that tunicamycin, an ER stress inducer, elevated ROS levels and diminished NO production in aortic rings, leading to impaired Ach-induced relaxation. Our current study demonstrated an increase in ROS production and inflammatory markers in DIO mice. Consequently, the inhibition of ER stress may represent a viable therapeutic strategy.

Extensive research has demonstrated that AMPK acts as a physiological suppressor of ER stress (Deng et al. 2024). Activation of AMPK has been shown to inhibit ER stress, enhance NO bioavailability, and improve vascular endothelial function in conditions such as pulmonary arterial hypertension and hypertension (Chen et al. 2019; Freitas Carvalho et al. 2019). In the present study, we found a downregulation of AMPKα in the aortae of DIO mice. Notably, natural products exhibit significant potential in the treatment of endothelial dysfunction, attributed to their pharmacological properties that inhibit ER stress, oxidative stress, and inflammatory pathways (Li et al. 2022). Celastrol, a pentacyclic triterpene, has demonstrated a beneficial protective effect in cardiovascular diseases through its anti-oxidative and anti-inflammatory properties (Ye et al. 2020). Celastrol inhibits Angiotensin II (Ang II)-induced cardiomyocyte hypertrophy and matrix protein deposition by binding to and inhibiting Signal Transducer and Activator of Transcription 3 (STAT3) (Deng et al. 2025). Furthermore, Celastrol attenuates lipopolysaccharide-induced acute lung injury by activating Nuclear Factor Erythroid 2–Related Factor 1 (Nrf1) and improving mitochondrial function (Feng et al. 2019). In this study, we found that celastrol attenuated ER stress and improved impaired endothelial function dependent on AMPK activation in DIO mice. Previous reports have indicated that celastrol exerts anti-obesity effects by enhancing leptin sensitization, decreasing food intake, and restoring glucose tolerance and insulin sensitivity (Zaric et al. 2020). Hyperlipidemia-induced intracellular generation of ROS has been implicated as a signaling mechanism contributing to endothelial dysfunction (Deng et al. 2024). In the present study, we observed that celastrol administration led to a reduction in body weight, blood lipid levels, and hepatic steatosis in DIO mice, which could be also responsible for ameliorating endothelial dysfunction. More importantly, we found that celastrol significantly reduce the level of ROS triggered by ER stress, including decreased iNOS and NOX2 levels, which may be a major factor in improving vascular endothelial function in DIO mice.

Some questions have arisen from our research. Firstly, what is the mechanism by which celastrol induces the activation of AMPKα? Previous studies have demonstrated that celastrol reduces the expression of Hepatic Sterol Regulatory Element-binding Protein 1c (Srebp-1c), enhances the phosphorylation of hepatic AMPKα, and mitigates lipid synthesis and metabolic damage in the liver (Zhang et al. 2017). It is plausible that Srebp-1c plays a role in the enhancement of endothelial function in DIO mice following celastrol treatment, a hypothesis that warrants further investigation. Additionally, the clinical application of celastrol as an oral therapeutic agent may be impeded by its low water solubility, narrow therapeutic window, and potential side effects (Xu et al. 2021). To address these challenges, modifications to its chemical structure and the development of novel pharmaceutical dosage forms are necessary.

## Conclusions

Our research demonstrates that celastrol enhances endothelial function in DIO mice by mitigating ER stress through the activation of AMPKα. Consequently, celastrol emerges as a promising candidate for the treatment of cardiovascular diseases and may also contribute to a deeper understanding of the disease's pathogenesis.

## Supplementary Information

Supplementary Material 1: Figure S1. Diet-induced obesity (DIO) mice and control mice were treated with saline or celastrol. DIO and control (Con) mice were treated with saline or celastrol (Cel, 100 μg/kg/day) intraperitoneally (i.p) for 8 weeks. (A) Schematic illustration of the experimental design. (B) Effect of celastrol on fat mass in DIO mice.

Supplementary Material 2. Figure S2. Effect of celastrol on the expression of upstream regulator and downstream effectors of ER stress in diet-induced obesity (DIO) mice. DIO and control (Con) mice were treated with saline or celastrol (Cel, 100 μg/kg/day) intraperitoneally (i.p) for 8 weeks. (A-D) The protein expression of BiP, ATF4, XBP1, and CHOP in aortae was analyzed by western blotting. Data are expressed as the means ± S.E.M (*n*=6/group), *P <0.05, one-way ANOVA followed by Newman-keuls post hoc test.

Supplementary Material 3. Figure S3. Effect of celastrol on LKB1 and ACC phosphorylated expression in diet-induced obesity (DIO) mice. DIO and control (Con) mice were treated with saline or celastrol (Cel, 100 μg/kg/day) intraperitoneally (i.p) for 8 weeks. The phosphorylation of LKB1 and ACC in the aortae was analyzed by western blotting. Data are expressed as the means ± S.E.M (*n*=6/group), *P <0.05, one-way ANOVA followed by Newman-keuls post hoc test.

## Data Availability

The datasets used and/or analysed during the current study are available from the corresponding author on reasonable request.
