# Supplementary figures and images for: Celastrol improves endothelial function in diet-induced obesity mice via attenuating endoplasmic reticulum stress through the activation of AMPK pathway
Source: Mol Med. 2025 Jun 11;31:233. doi: 10.1186/s10020-025-01259-6 (PMC12153150; doi:10.1186/s10020-025-01259-6)

## Supplemental Figure S1

A

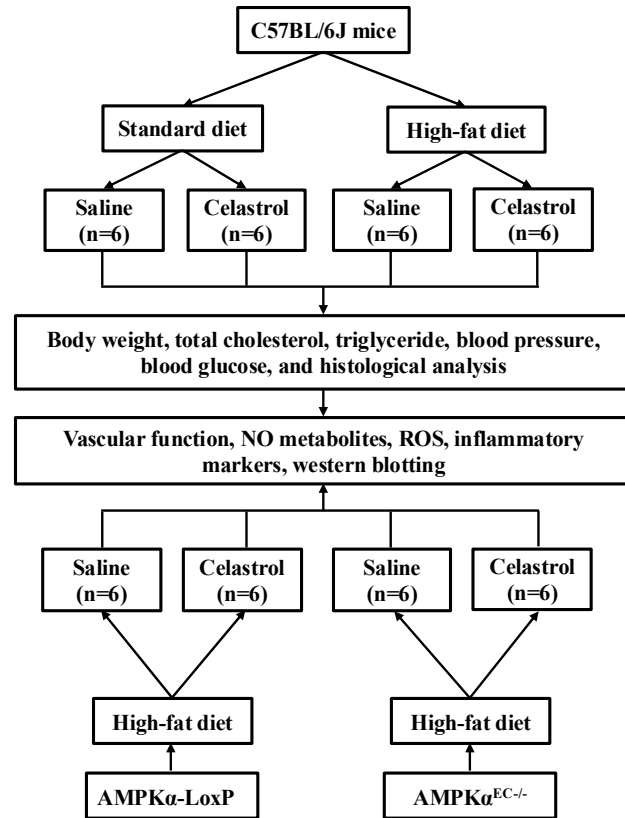

# B

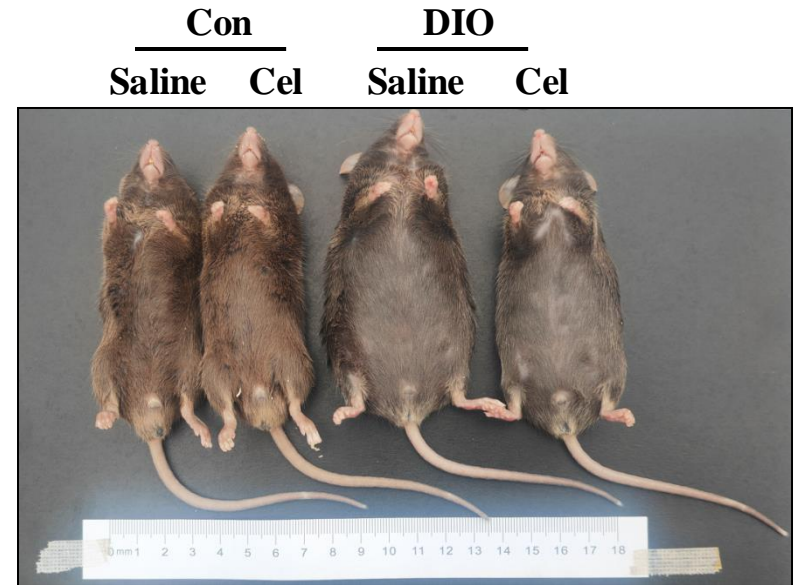

Supplement: Supplementary file 1 — Supplementary Material 1: Figure S1. Diet-induced obesity (DIO) mice and control mice were treated with saline or celastrol. DIO and control (Con) mice were treated with saline or celastrol (Cel, 100 μg/kg/day) intraperitoneally (i.p) for 8 weeks. (A) Schematic illustration of the experimental design. (B) Effect of celastrol on fat mass in DIO mice. [file 10020_2025_1259_MOESM1_ESM.pdf]

# Supplemental Figure S2

A

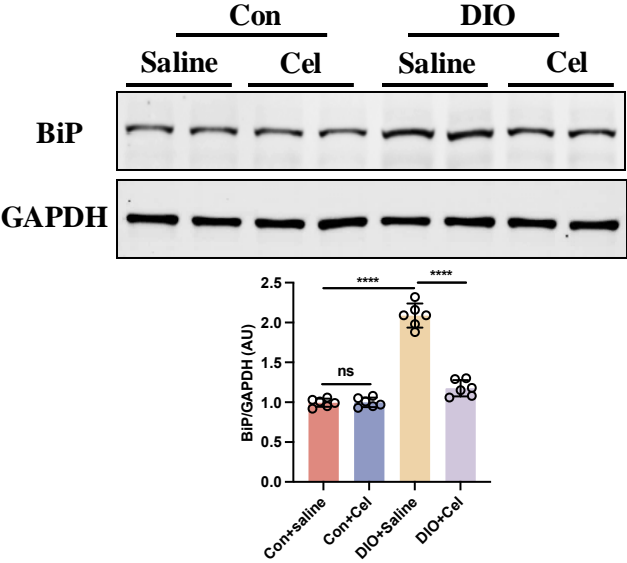

B

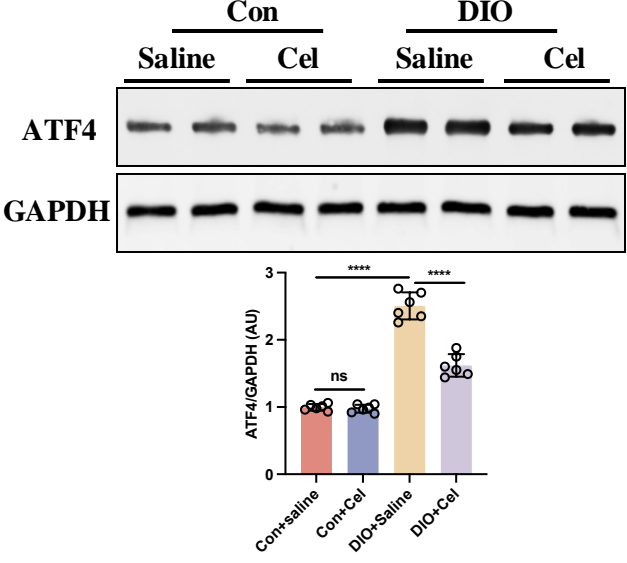

C

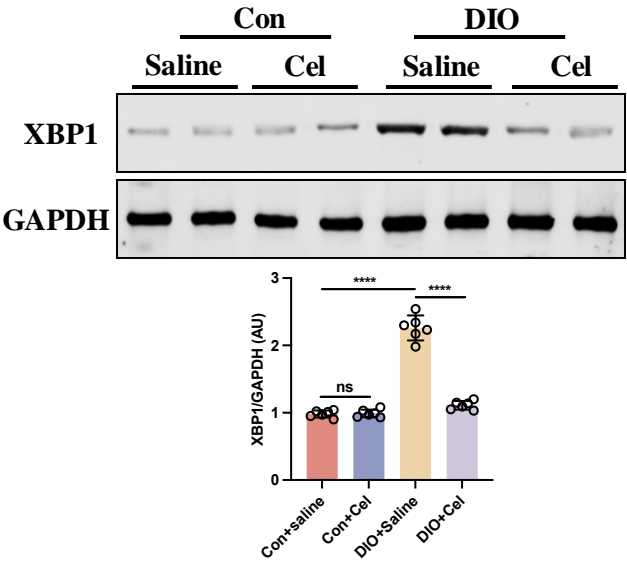

D

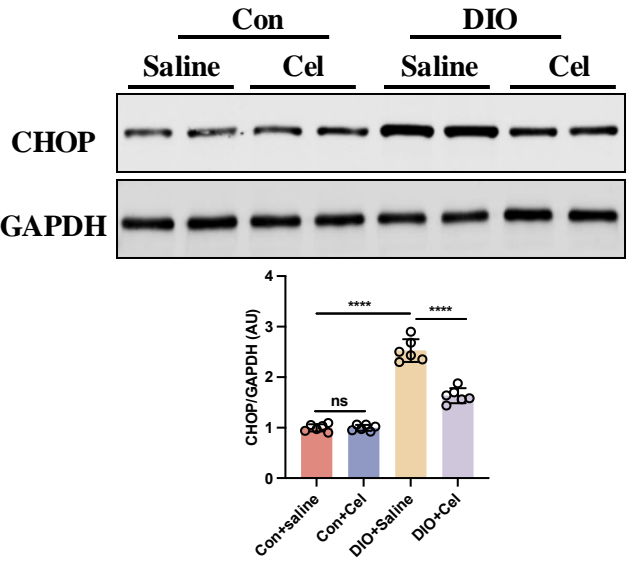

Supplement: Supplementary file 2 — Supplementary Material 2. Figure S2. Effect of celastrol on the expression of upstream regulator and downstream effectors of ER stress in diet-induced obesity (DIO) mice. DIO and control (Con) mice were treated with saline or celastrol (Cel, 100 μg/kg/day) intraperitoneally (i.p) for 8 weeks. (A-D) The protein expression of BiP, ATF4, XBP1, and CHOP in aortae was analyzed by western blotting. Data are expressed as the means ± S.E.M (n=6/group), *P <0.05, one-way ANOVA followed by Newman-keuls post hoc test. [file 10020_2025_1259_MOESM2_ESM.pdf]

Supplemental Figure S3

A

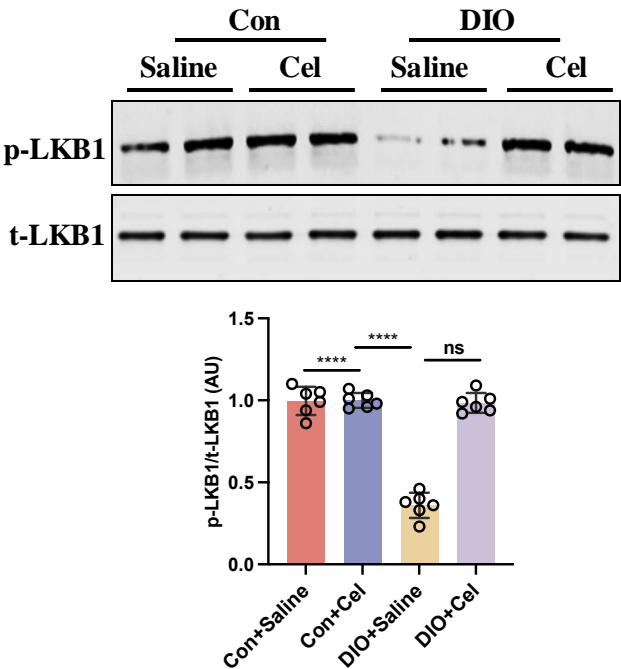

B

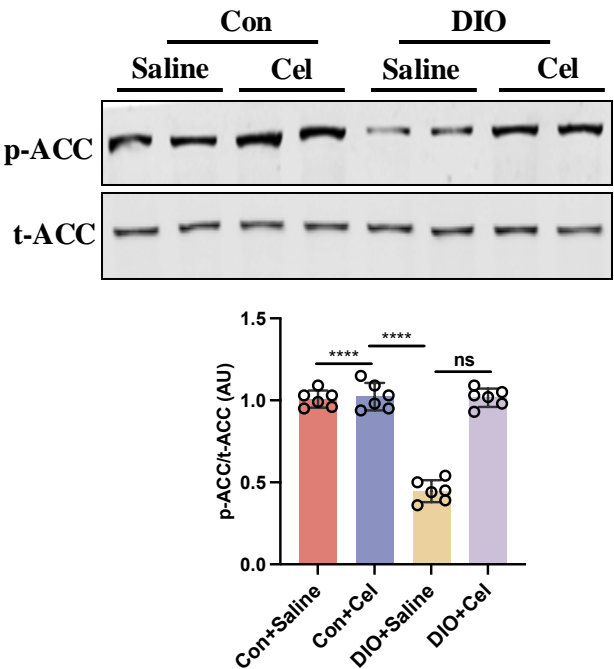

Supplement: Supplementary file 3 — Supplementary Material 3. Figure S3. Effect of celastrol on LKB1 and ACC phosphorylated expression in diet-induced obesity (DIO) mice. DIO and control (Con) mice were treated with saline or celastrol (Cel, 100 μg/kg/day) intraperitoneally (i.p) for 8 weeks. The phosphorylation of LKB1 and ACC in the aortae was analyzed by western blotting. Data are expressed as the means ± S.E.M (n=6/group), *P <0.05, one-way ANOVA followed by Newman-keuls post hoc test. [file 10020_2025_1259_MOESM3_ESM.pdf]
